# Progress in Gynæcology

**Published:** 1896-06-20

**Authors:** 


### Progress in Gynæcology.

**Cancer of the Uterus.**—Dr. Cordier,[1] in a paper on the removal of the uterus for cancer draws the following deductions: (i.) Cancer of the cervix uteri, if left without surgical interference, always kills; (ii.) the disease is in most instances primarily a local process; (iii.) early hysterectomy will cure quite a percentage of these cases; (iv.) the microscope, while a great diagnostic aid, is not infallible in its findings; (v.) the experienced surgeon is warranted in resorting to hysterectomy, even in doubtful cases; (vi.) every malignant gravid uterus should be removed before the disease has advanced beyond the period of a probable cure.

Dr. Dunning[2] states that if he should find hardening of the cervix and the development of a neoplasm with the presence of discharges, sanguineous or offensive in character, even though the microscope failed to reveal positively the existence of malignant disease, he should be inclined to remove the uterus. As to the advisability of removing the cervix alone, it might be done if the surgeon was certain that the epitheliomatous variety existed; yet the woman's life was in peril after the neoplasm was removed, because of the scar tissue which remained. In proof of this Dr. Dunning cited cases.

Dr. Wiggin[3] relates a case of a woman aged thirty-eight with five months' history of fetid discharge accompanied by increased menstruation, and the patient had become cachectic. Examination had shown the uterus enlarged and the cervix indurated. The specimen of tissue removed from the uterus with a curette proved to be a superficial carcinoma. After hysterectomy examination of the uterus showed the carcinoma to be very superficial and limited in extent, and indicated that the disease was in its incipiency. The case is very instructive as showing the importance of being on the alert for malignant disease in the early stages. Dr. Wiggin thinks if there is any doubt about the malignant nature of a growth the patient should have the benefit of the doubt, and the uterus should be extirpated. In operating he uses the Makenrodt incision, beginning about one-third of an inch below the meatus and incising the anterior wall of the vagina, dissecting both flaps laterally. This method seems to give a much larger field for operation, and hence makes it much easier to tie the ovarian arteries and to avoid the uterus.

Dr. Barrow[4] says vaginal hysterectomy appeals strongly to the gynæcologist on account of the comparative freedom from shock and the rapidity of convalescence; but for cases of carcinoma, it seemed to him that the operation by the vagina was an imperfect one. In the vaginal operation the uterine arteries are ligated close to the uterus, leaving behind the contents of the pelvis, except the uterus and sometimes the tubes and ovaries. Two years ago a mode of procedure was brought before the Obstetrical Society, which seemed to hold out better prospects of success than vaginal hysterectomy — suprapubic hysterectomy with ligation of the uterine arteries outside of the ureters and close to the pelvic wall. In this way the malignant disease could be removed from the pelvis as thoroughly as it could be removed in operations for carcinoma of the mammary gland. The folds of the broad ligament are separated until the internal iliac artery is reached, where it divides into the anterior and posterior branches. If the ovarian and superior vesical arteries are tied outside of the ureter, close to their emergence from the anterior branch of the internal iliac artery, the field of operation in the pelvis is perfectly bloodless. It is entirely unnecessary to introduce a stylet into the ureters to one familiar with the anatomy of this region, and if it is done there is the danger of causing inflammation of the ureters.

**Epithelioma of Cervix Uteri in a Woman aged 23.**—Bondin[5] publishes this case owing to its extreme rarity in one so young. The catamenia appeared at 13, she was married at 15, and was delivered of a living child when only 17 years of age. Seven months before she was seen the periods became profuse, then intermittent hæmorrhages, with a sero-sanguineous non-fetid discharge, appeared; clots and débris were passed. The patient was emaciated and pale, with anæmic mucous membranes. Vaginal examination revealed a soft, irregular, fungous, easily-bleeding mass at the fundus of the vagina. The cul-de-sacs were quite free; the cervix could not be distinguished. Supra-vaginal amputation was performed, and six weeks later she had lost all her hæmorrhages, was making flesh, and generally improving. Bondin considers that epithelioma of the cervix is exceedingly rare in women below the age of 30. Cases of malignant disease in these cases are more frequently sarcoma of the decidua. The histological examination in this case was made by a competent observer.

**Primary Malignant Disease of the Clitoris.**—Crumston[6] states that this neoplasm usually occurs after the menopause. Former labours, trauma, syphilitic lesions, psoriasis, eczema of the vulva, and vaginal discharges are given as the predisposing causes. The affection appears in all classes of society, though mainly the result of poverty and vice. Its development is insidious, the patient subsequently complaining of heat and burning at the vulva. Again, a pruritus vulvæ is often an early sign, accompanied by more or less fetid discharge. Upon examination a tumour or an ulceration is detected—the former more frequently—which proves to be a nodular type of epithelioma. At first limited to the clitoris, the growth tends to invade the neighbouring parts. The inguinal glands are sometimes involved. Simple or melanotic sarcoma, as also myxosarcoma, of the clitoris have also been occasionally met with. These sarcomata are usually rapid in growth. Complete excision or dissection by means of the knife and thermo-cautery, preferably the former, is the line of treatment advised.

**Vaginal Cœliotomy.** — Taylor[7] writes the new operation of vaginal cœliotomy or anterior colpotomy is attracting considerable attention among gynæcologists of all countries. It is, perhaps, not so much a new operation as a new "route" to the female pelvis and abdomen—a route by which many operations may be carried out on the uterus and its appendages without causing any visible wound or abdominal cicatrix—a route which has its special advantages and disadvantages, but, in spite of the latter, seems likely to hold its own as a permanent addition to surgical methods. In vaginal cœliotomy, instead of approaching the pelvis through the abdominal wall, the pelvic cavity is opened between the uterus and the bladder. Taylor advises that the operation be carried out in a strictly antiseptic manner. With an Arward's speculum resting on the posterior vaginal wall, the anterior lip of the cervix is seized with volsella and drawn down to the vulva. A metal sound of fair size (nine or ten) is passed into the bladder, and after turning the point down to mark the lower limit of the bladder, the beak is reversed and held up by an assistant, while the operator makes a transverse incision about the line where the anterior vaginal wall joins the cervix. The centre of the upper border of the incision is then seized with volsella and drawn sharply upwards by the assistant, so as to draw vaginal wall and bladder away from the cervix. The incision is now deepened and enlarged by scissors, the connective tissue between the cervix and bladder is opened, and the bladder is bluntly separated from the anterior aspect of the cervix by the finger. As this is done the sensitive part of the finger, being applied to the anterior uterine wall, usually becomes conscious of a thin membrane sliding between the finger and the uterus at the highest and deepest part of the wound. This is the anterior fold of peritoneum between the uterus and the bladder, and this fold may be brought to the surface of the incision, or so near to the surface, that it may be seized by catch forceps, and the fold then opened. As soon as the peritoneal fold is opened the opening is enlarged by the fingers, and the highest accessible point of the anterior uterine wall is seized with volsella or by a provisional silk suture passed through the wall and drawn forwards and downwards. The cervix is now pushed back, and by this double action the uterus is turned or twisted forwards, and in favourable cases the fundus may be pulled quite out of the incision at once. The delivery of the fundus out of the incision is a necessary preliminary to any operation on the uterine appendages, and sometimes, especially when the uterus is large, it is a matter of some difficulty. The size of the uterus is an important factor, and should be carefully estimated before the operation is begun.

Dr. Kineberg[8] reports forty-two vaginal sections with only one death, and that from sepsis, which, he thinks, originated from improper disinfection of the vagina. He considers a longitudinal incision best because—(1) it gives more room, (2) the ureters are not likely to be injured, (3) there is less hæmorrhage, and (4) it furnishes the best method of performing uterine fixation.

**The Treatment of Pelvic Exudations in Women.**—The "Revue Internationale de Médécine et de Chirurgie"[9] publishes an extract, saying that when a pelvic exudation follows confinement, or results from an infection after operation, we should at first restrict ourselves to a purely medical treatment. Under the influence of this the inflammatory symptoms will diminish gradually, and frequently the exudation will completely disappear. Often, however, it persists, and resists medical treatment. In this case it becomes a question of operative treatment, and this is indicated in all cases in which the fever and exudation do not disappear after several weeks of treatment. If pus accumulates in Douglas's cul-de-sac, it is in this region that the incision should be made, and the tissues carefully divided layer by layer.

The blood vessels of the vagina and of the purulent sac often give rise to an abundant hæmorrhage, which must be arrested before going on with the operation. The sac is then to be washed out with a solution of boric acid and salicylic acid, and tamponed with iodoform gauze.

If the exudation takes place between the abdominal walls and the peritoneum, the incision should be made carefully from two to three finger-breadths above Poupart's ligament, and parallel with it. After the abscess is opened, the cavity is to be washed out and tamponed.

Sometimes, says the author, the situation of the purulent collection cannot be exactly determined, and we are then obliged to substitute laparotomy by extra-peritoneal incision; but this, he says, is rare. It is important to evacuate the pus or the serosity, and to disinfect the cavity, for, according to Buschbeck[10] and Ettinger,[10] this procedure suppresses the fever, and leads more or less rapidly to a cure.

[1] New York Medical Journal. April 18, 1896. [2] New York Medical Journal, April 18, 1896. [3] New York Medical Journal, March 28, 1896. [4] New York Medical Journal, March 28, 1896. [5] Practitioner, April, 1896. [6] Annals of Gynæcology and Pediatrics. 1896, No. 5, p. 268. [7] Birmingham Medical Review, February. 1896. [8] Medical Week, March 20, 1896. [9] New York Medical Journal, April 11, 1896. [10] Semaine Medicale, March 25, 1896.

